# Hemospray Versus Conventional Therapy for Non-variceal Upper Gastrointestinal Bleeding: A Systematic Review and Meta-Analysis

**DOI:** 10.7759/cureus.55079

**Published:** 2024-02-27

**Authors:** Mihir P Shah, Sania Saleem, Bashar Attar, Can Cui, Hemant Mutneja

**Affiliations:** 1 Internal Medicine, John H. Stroger, Jr. Hospital of Cook County, Chicago, USA; 2 Gastroenterology and Hepatology, Rush University Medical Center, Chicago, USA; 3 Gastroenterology and Hepatology, John H. Stroger, Jr. Hospital of Cook County, Chicago, USA; 4 Gastroenterology, John H. Stroger, Jr. Hospital of Cook County, Chicago, USA

**Keywords:** systematic review and meta-analysis, conventional endoscopic therapy, non-variceal upper gastrointestinal bleeding, hemostatic powder spray tc-325, tc-325, hemostasis, hemospray

## Abstract

Hemospray (TC-325; Cook Medical, Winston-Salem, NC) has been used effectively in hemostasis in non-variceal upper gastrointestinal (GI) bleeding. Current guidelines suggest using Hemospray as a temporizing measure or adjunct technique. This systematic review and meta-analysis aimed to evaluate the efficacy and safety of Hemospray as a modality for primary hemostasis.

We searched MEDLINE, CENTRAL, and CINAHL (Cumulative Index of Nursing and Allied Health Literature) databases from inception to August 1, 2022. Three independent reviewers performed a comprehensive review of all original articles describing the application of Hemospray as the primary method of hemostasis in non-variceal upper GI bleeding patients. Three reviewers independently reviewed and abstracted data and assessed study quality using the Cochrane risk of bias tool. Primary outcomes were (1) primary hemostasis rate, (2) rebleeding rate until hospital discharge or death, (3) need for surgery, and (4) overall mortality rate.

Of the 211 studies identified, 146 underwent title and abstract review, and four were included in the systematic review. Pooled results from 303 patients showed that compared to standard of care, Hemospray has significantly higher odds of primary hemostasis (OR: 3.48, 95% CI: 1.09-11.18, p = 0.04). There was no statistically significant difference in terms of rebleeding rates (OR: 0.79, 95% CI: 0.24-2.55, p = 0.69), need for surgery (OR: 1.62, 95% CI: 0.35-7.41, p = 0.54), or overall mortality (OR: 1.08, 95% CI: 0.56-2.08, p = 0.83).

This systematic review and meta-analysis prove that Hemospray is a better modality of primary hemostasis in non-variceal upper GI bleeding when used as a primary method. At the same time, there is no significant difference in complications, including rebleeding, need for surgical intervention, and all-cause mortality.

## Introduction and background

Non-variceal acute upper gastrointestinal (GI) bleeding is a common medical emergency that results from various etiologies [1]. The most common cause of non-variceal upper GI bleeding is peptic ulcer disease, while malignancy also plays an important role [2]. Traditionally, the standard treatment of non-variceal bleeding has been endoscopic hemostatic measures. Metallic clips, adrenaline injections, and thermal probes have been widely used for GI bleeding secondary to peptic ulcer disease [3,4]. Argon plasma coagulation is the major treatment option for malignancy-related GI bleeding and diffuse vascular lesions [5]. TC-325 (Hemospray, Cook Medical, Winston-Salem, NC) is a proprietary hemostatic powder that can be sprayed directly to the active bleeding lesion. It has an adsorptive feature and provides a barrier at the bleeding site. Moreover, the easy application of Hemospray to anatomical difficult areas is a major advantage compared to conventional techniques of endoscopic hemostasis. Multiple studies have reported that Hemospray has a comparable immediate hemostasis rate compared to conventional endoscopic treatment. However, these studies are mostly limited by small sample size. A systematic review comparing Hemospray monotherapy versus a combination of Hemospray and conventional techniques concluded that Hemospray had significantly high rebleeding rates [6]. Another systematic review also compared the efficacy and re-bleeding rate of adding Hemospray to conventional therapy [7]. Current guidelines recommend Hemospray as a stopgap or adjunct technique, but the use of Hemospray as the primary method of hemostasis has not been well studied [3,4]. We present a systematic review and meta-analysis of randomized clinical trials comparing primary hemostasis, rebleeding rates, mortality, and need for surgery in patients who received Hemospray as a primary hemostatic measure versus conventional endoscopic hemostasis in non-variceal acute upper GI bleeding.

## Review

Method

Our study was performed in accordance with the Preferred Reporting Items for Systematic Reviews and Meta-Analyses (PRISMA) statement and Cochrane guidelines for systematic reviews [8,9].

Search Strategy

The search strategy was designed and conducted by the authors. Two reviewers independently and in duplicate searched MEDLINE, CENTRAL, and CINAHL (Cumulative Index of Nursing and Allied Health Literature) using a pre-defined protocol and multiple search terms ("hemostatic powder," "Hemospray," "TC-325," "mineral powder") from inception to August 1, 2022. All titles and abstracts were identified by the authors and screened to accrue potentially eligible studies. The search was restricted to studies on human subjects in English. Then, the same reviewers independently assessed all selected full-text manuscripts for eligibility. Duplicate studies were filtered using Zotero reference management software. Disagreements between the two reviewers were resolved through consensus and after input from the third reviewer and principal investigator.

Eligibility Criteria

The population of interest was adult patients (age > 18 years) presenting with non-variceal upper GI bleeding. The specific inclusion criteria for the systematic review and meta-analysis were: (i) all randomized trials comparing Hemospray to the standard of care (mechanical clipping, thermal cautery, injection therapy) for primary hemostasis of non-variceal upper GI bleeding; (ii) studies with information available to evaluate the hemostasis, rebleeding, and mortality rates based on the management strategy used; (iii) full-text articles available in the English language. Thus, reviewed studies included in our analysis were randomized trials comparing the hemostasis rates with Hemospray and standard-of-care therapy. Non-randomized studies and studies evaluating Hemospray as salvage therapy were excluded from the analysis.

Study Characteristics and Quality Assessment

We selected data collection forms for randomized trials based on the Cochrane Collaboration risk assessment tool to adhere to principles of sound methodological quality. For each study, we ascertained seven domains to identify imbalances in baseline characteristics. We used the terms “low risk” and “high risk” of bias at the study level for the scoring system. In our study, “unclear bias” was judged from baseline imbalance, which could not be ascertained from the seven domains. Quality assessments were also conducted independently, and discrepancies were resolved by consensus. There was some degree of heterogeneity in the included studies, especially concerning the sub-types of the patient population the interventions were assessed for, as well as some differences in the intervention itself. The heterogeneity was further assessed statistically and is dissected in detail in the discussion section.

Outcome Measures

The outcomes for this systematic review and meta-analysis were as follows: (i) primary outcomes: primary hemostasis rate; (ii) secondary outcomes: rebleeding rate (until hospital discharge or death), need for surgery, and overall mortality rate.

Data Extraction

Three reviewers independently reviewed and abstracted data on hemostasis, rebleeding, and mortality rates for each eligible study. If there were multiple reports stemming from a specific study database, data from the most robust study were extracted with other studies contributing toward the bibliography. The reviewers sorted the data separately in all stages of study collection, data extraction, and quality assessment. All discrepancies found between reviewers were resolved with consensus and input from other authors.

Quantitative Data Analysis

All data were analyzed using the computer software RevMan 5.4.1 version 5.4.1 (Cochrane Collaboration, London, UK). The final pooled risk estimates were obtained using random effects models by the methods of DerSimonian and Laird with inverse variance weighting [10]. Raw data for detection and participation events and nonevents from each study were used to calculate a crude odds ratio (OR) for each study. The Cochrane Q and the I2 statistics were calculated to assess heterogeneity between studies. P < 0.10 for the chi-square test and I2 <20% were interpreted as low-level heterogeneity.

Results

Results of the Search

**Figure 1:**
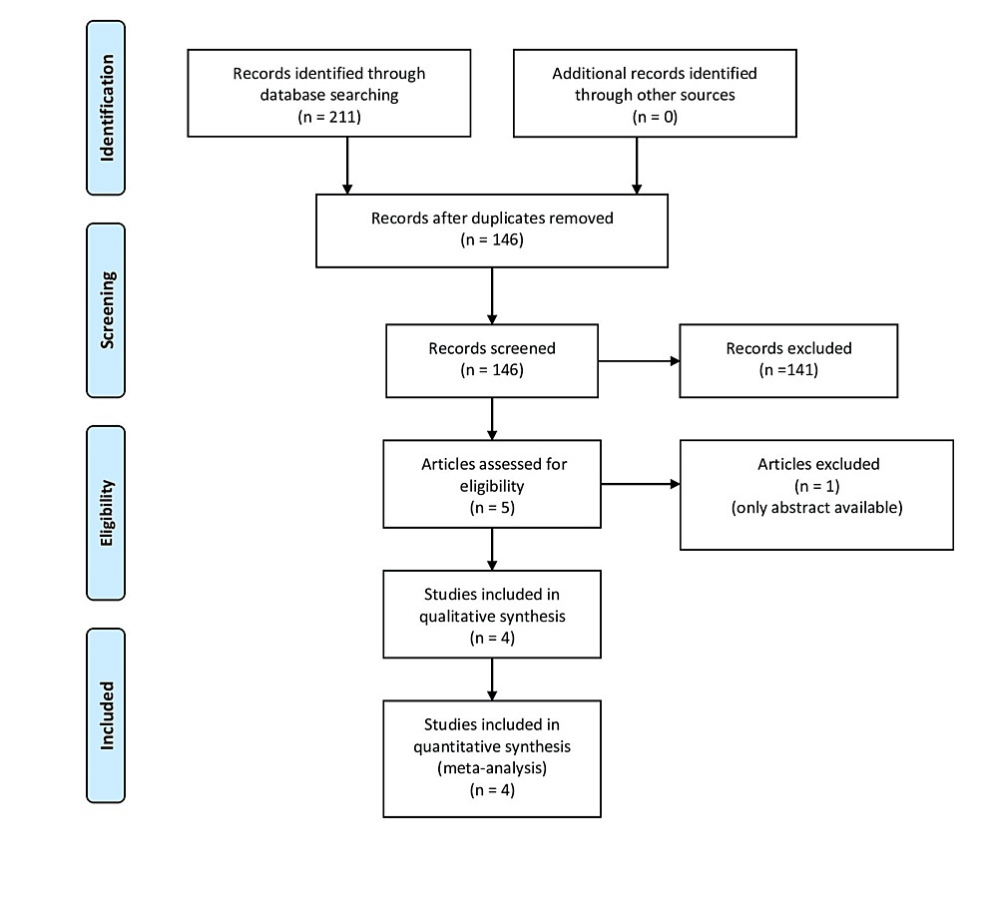
PRISMA flow diagram PRISMA: Preferred Reporting Items for Systematic Reviews and Meta-Analyses.

The initial library search identified 211 potentially relevant citations from PubMed, CINAHL, and CENTRAL. Subsequently, after the removal of duplicates, 146 underwent title and abstract review. The remaining manuscripts were scrutinized further and finally, four studies were included in the full review. There was no overlap of patients among the different studies. The PRISMA flowchart for the search strategy is shown in Figure 1.

Included Studies

Four studies with a total of 303 patients were included in the review. All the studies were randomized, controlled clinical trials. The characteristics of the included studies are shown in Table 1.

**Table 1:** Characteristics of included studies RCT: randomized controlled trial; UGIB: upper gastrointestinal bleeding; GI: gastrointestinal; TC-325: Hemospray.

Study	Type of study	Location	Duration of study	No. of patients	Patient population	Comparison
Baracat et al. (2020) [11]	Pilot single-center RCT	Sao Paulo, Brazil	2015-2017	39	All patients with a history of UGIB or in-hospital suspected UGIB	TC-325 + epi injection vs. endoscopic clipping + epinephrine injection
Kwek et al. (2017) [12]	Prospective RCT	Singapore	2013-2015	20	Peptic ulcer with high-risk stigmata of recent hemorrhage (Forrest IA, IB, IIA, IIB)	TC-325 vs. endoscopic clipping + epinephrine injection
Chen et al. (2020) [13]	Pilot prospective RCT	Montreal, Canada	2014-2016	20	Malignant GI bleeding	TC-325 vs. endoscopic clipping + epinephrine injection
Lau et al. (2022) [14]	One-sided, non-inferiority RCT	Hong Kong, Thailand, and Singapore	2015-2018	224	Acute upper GI bleed (non-variceal, Forrest 1A or 1B)	TC-325 vs. endoscopic clipping + epinephrine injection

Risk of Bias in Included Studies

**Figure 2:**
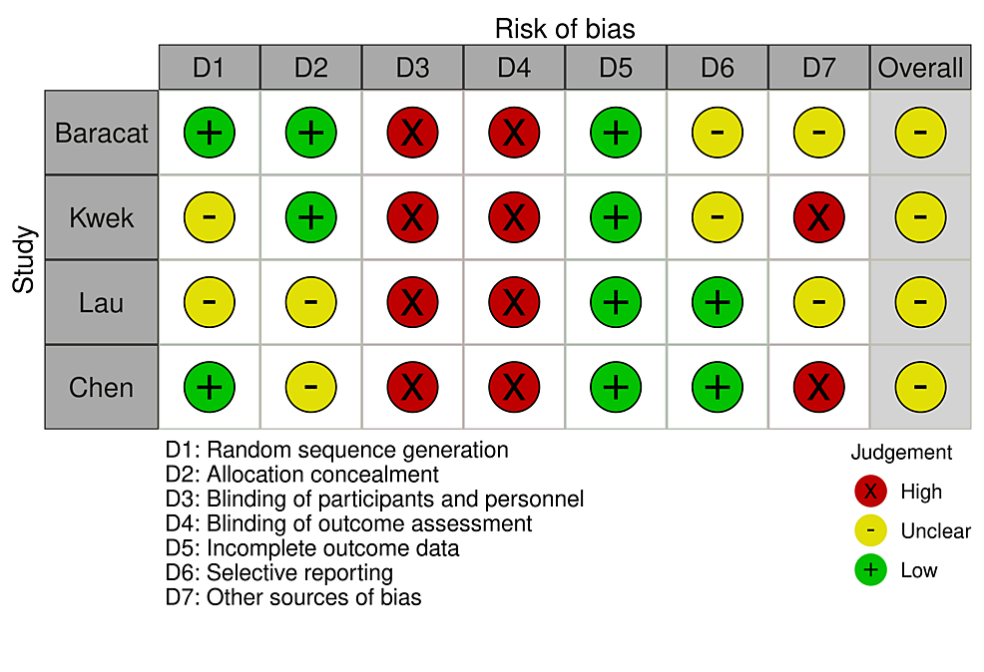
Risk of bias traffic light plot Baracat et al. [11], Kwek et al. [12], Lau et al. [14], and Chen et al. [13].

**Figure 3:**
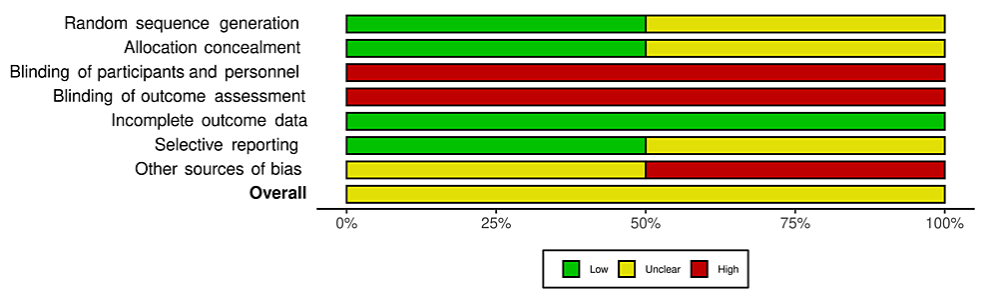
Risk of bias summary plot

Due to the nature of the studies, blinding of participants and personnel (e.g., endoscopists) was not possible, hence all the studies had a high risk of performance and detection bias. While Baracat et al. [11] had a low risk of selection bias, for the other studies, the risk was unclear. Additionally, for Kwek et al. [12] and Chen et al. [13], there were limitations concerning patient selection (Kwek et al. [12]: limited to bleeding secondary to GI malignancies; Chen et al. [13]: limited to bleeding secondary to peptic ulcer disease). However, all the studies were at low risk for attrition bias, with no reported loss of patients to follow-up. Overall, the studies were fair with regard to the risk of bias. Due to the low number of included studies (n < 10), our meta-analysis is underpowered to detect any publication bias. The quality assessment is illustrated in Figures 2, 3.

Outcome Analysis

**Figure 4:**
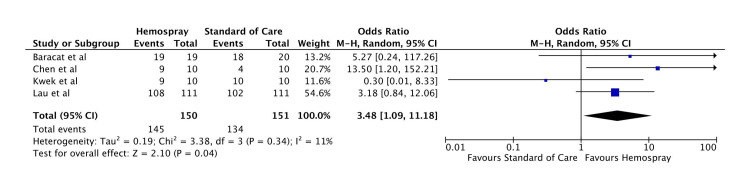
Forest plot for primary hemostasis rate Baracat et al. [11], Kwek et al. [12], Chen et al. [13], and Lau et al. [14].

Primary hemostasis rate: The odds of primary hemostasis were significantly higher with Hemospray compared to standard of care (OR: 3.48, 95% CI: 1.09-11.18, p = 0.04). The forest plot for this analysis is shown in Figure 4.

**Figure 5:**
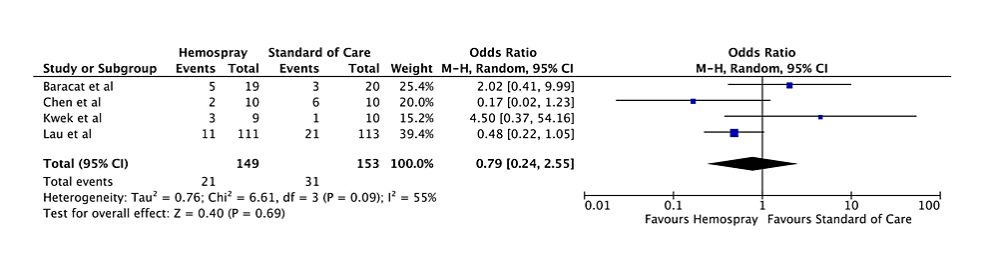
Forest plot for rebleeding rate Baracat et al. [11], Kwek et al. [12], Chen et al. [13], and Lau et al. [14].

Rebleeding rate: There was no statistically significant difference between Hemospray and standard of care in terms of rebleeding rates (OR: 0.79, 95% CI: 0.24-2.55, p = 0.69). The forest plot for this analysis is shown in Figure 5.

**Figure 6:**
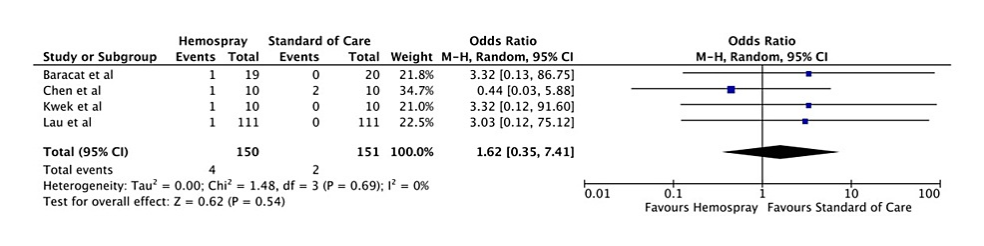
Forest plot for the need for surgery Baracat et al. [11], Kwek et al. [12], Chen et al. [13], and Lau et al. [14].

Need for surgery: There was no statistically significant difference between Hemospray and standard of care in terms of the need for surgery (OR: 1.62, 95% CI: 0.35-7.41, p = 0.54). The forest plot for this analysis is shown in Figure 6.

**Figure 7:**
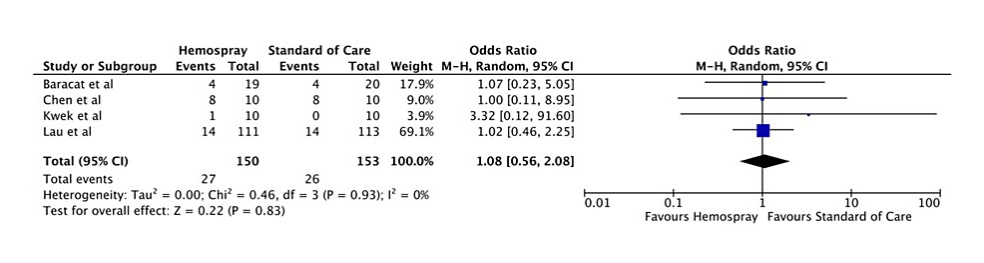
Forest plot for overall mortality Baracat et al. [11], Kwek et al. [12], Chen et al. [13], and Lau et al. [14].

Overall mortality: The overall mortality rate was not statistically different amongst the two groups (OR: 1.08, 95% CI: 0.56-2.08, p = 0.83). The forest plot for this analysis is shown in Figure 7.

Discussion

Our study demonstrates that compared to the standard of care, the odds of primary hemostasis with Hemospray were significantly higher, and the result was statistically significant. This was accompanied by non-significant differences in rebleeding rate, need for surgery, and overall mortality.

GI hemorrhage is the most common cause of GI-related admissions in the United States with 507,440 admissions in 2012 as per the Nationwide Inpatient Sample (NIS), with a mortality of 2%. Upper GI bleeds have an incidence in various epidemiological studies ranging from 48 to 160 cases per 100,000 individuals per year [15,16]. The most common causes of non-variceal upper GI bleeding include peptic ulcers, Mallory-Weiss tears, malignant ulcers, erosive esophagitis, Dieulafoy lesions, vascular ectasias, etc. [17,18]. The current recommendation is endoscopic therapy within 24 hours of admission for upper GI bleeding and hemostatic therapy to be applied to actively spurting or oozing bleed and non-bleeding visible vessels [3]. Endoscopic hemostatic measures commonly in practice are thermal contact devices (bipolar electrocoagulation and heater probe), absolute ethanol injection, hemoclips, argon plasma coagulation, and soft monopolar electrocoagulation [3]. TC-325 is a biologically inert powder that is deployed using a Hemospray device. It is a relatively newer modality that is deployed to achieve endoscopic hemostasis for the management of GI bleeding. Hemospray device is made up of three parts: an application catheter, a carbon dioxide (CO2) cartridge, and a syringe containing TC-325 powder. First, the CO2 cartridge is activated. Then the bleeding site is identified, the adjacent area is flushed, and blood is removed. Next, the Hemospray catheter is advanced through the access catheter after flushing while making sure to avoid contact with blood in tissues. The powder is then deployed using a trigger button, applying continuously in short bursts until the site is completely covered and no active bleeding is visualized. TC-325 works by providing a mechanical barrier at the bleeding site after meeting an aqueous medium. TC-325 powder enhances clot formation in vivo and shortens coagulation in vitro [19]. It is a nonthermal, non-contact, and nontraumatic modality of hemostasis and is, therefore, less likely to cause complications, including perforation. It is easier to apply as a direct spray to the bleeding spot located in the anatomical difficult areas. With its great safety profile, great interest has been rising to ascertain its efficacy in acquiring hemostasis and to determine whether it improves outcomes, including primary hemostasis, mortality, rebleeding rates, or the need for further surgical intervention. Previously, the American College of Gastroenterology (ACG) guidelines suggested the use of TC-325 as a temporizing measure that should be followed by a second definitive hemostatic modality. The main concern was the higher chances of rebleeding as Hemospray is ultimately sloughed off from mucosa. Higher rebleeding rates were observed in many observational studies of TC-325, e.g., 31% (95% CI: 26%-37%) in a meta-analysis of 18 observational studies and two randomized controlled trials (RCTs) [6]. By contrast, the results from Lau et al. suggested that Hemospray may have a better primary bleeding control with no difference in mortality or rebleeding rates [14]. A systematic review and meta-analysis by Aziz et al. [20] was recently published in 2020, which demonstrated a high technical (97%) and clinical (91%) success rate in controlling upper GI bleed.

The main limitation of the meta-analysis was the inclusion of non-randomized studies, which introduced selection bias in the study. Another limitation was the inclusion of studies in which Hemospray was used in conjunction with conventional therapy or as a rescue modality after the failure of hemostasis using other modalities, which may have introduced some clinical heterogeneity. Most cases in this meta-analysis used Hemospray as the final hemostatic agent or rescue therapy. Our study is one of the first meta-analyses done on randomized control trials whereby outcomes were assessed when Hemospray was used as a primary hemostatic measure as compared with conventional modalities. Our analysis shows that Hemospray has a better primary hemostasis rate when used as a primary therapy alone as compared to combined conventional therapy. Also, there was no statistically significant difference in rebleeding rates, need for surgical intervention, or mortality between the two groups. Previous studies have proven its efficacy to be used as a rescue measure for hemostasis when other methods have failed. Hemospray also improved cost-effectiveness when used in combination with other techniques [21]. Multiple factors are postulated to be contributory to the Hemospray’s advantage. Its easy applicability to difficult anatomic positions, such as lesser curvature gastric body and posterior wall of the duodenal bulb, has been well recognized. These areas are generally harder to reach and to apply therapies that require precise localization of lesions, to be deployed and be effective (including hemoclip, heater probes, etc.). It has been proven to have great efficacy in treating diffuse mucosal bleeds and bleeds related to GI malignancies [22]. The lesser need for expert endoscopic skills looks to be one of its great advantages if it is applied universally. A systematic review by Mutneja et al. showed that the pooled rebleeding rate was 14% in the patients who received Hemospray [23]. Aziz et al. showed that rebleeding rate following the use of Hemospray was seen in 24% of patients compared to 19% in the control group, but that most likely would be biased as it contained trials in which Hemospray was used as rescue therapy, hence likely included lesions with already difficult hemostasis [20]. ​​​​​​Our meta-analysis demonstrated no significant difference in mortality or need for surgical intervention among the two groups. Our study is underpowered to determine any significant difference, if any. The main strength of our study is that it includes data from randomized control trials decreasing the selection bias. Importantly, it includes studies done on different demographic populations, including Singapore, Thailand, Hong Kong, and Canada, which helps with the generalizability of its results and its application worldwide. One limitation of our meta-analysis is the clinical heterogeneity among the population of RCTs. While Kwek et al. [12] studied Hemospray versus conventional combined technique exclusively in patients with peptic ulcer bleeding, Baracat et al. [11] and Lau et al. [14] included patients with all causes of non-variceal GI bleeding, of which malignancy-associated GI bleeding composed 13% and 14.3% of the cases, respectively. Chen et al.'s [13] study was done exclusively on patients with malignancy-associated GI bleed. In Baracat et al.'s study, epinephrine injection was used in the Hemospray group, which introduces interventional heterogeneity. Another major limiting factor is the inability to achieve complete blinding. Treating endoscopists were unable to be blinded because of the need for documentation and deploying hemostasis measures.

Our study did not have a sensitivity analysis or a sub-group analysis. The small number of total studies and the overall patient population of 303 may have led to limited power in the statistical analysis. Also, given the small number of studies included in the analysis, a funnel plot assessing publication bias was not done.

## Conclusions

In summary, our analysis strongly suggests that Hemospray is a better modality in terms of achievement of primary hemostasis as compared with conventional endoscopic therapy. There is no statistically significant difference between the rebleeding rate, need for surgery, and overall mortality between the two modalities. Since it requires less precision and endoscopic skill, it is an up-and-coming modality to be studied and employed as a primary method in the future. This study reinforces the need for further research to validate our findings. We recommend that more randomized control trials should be undertaken so that more data are available to better define the outcomes of Hemospray as a primary hemostatic measure, including rebleeding within 72 hours, mortality within 30 days, need for ICU admission, need for angiographic embolization, length of stay, and cost of hospitalization. Further economic analyses can also be made to determine the cost threshold to use Hemospray as a primary hemostasis measure.
